# Prediction of Hospitalization due to Adverse Drug Reactions in Elderly Community-Dwelling Patients (The PADR-EC Score)

**DOI:** 10.1371/journal.pone.0165757

**Published:** 2016-10-31

**Authors:** Nibu Parameswaran Nair, Leanne Chalmers, Michael Connolly, Bonnie J. Bereznicki, Gregory M. Peterson, Colin Curtain, Ronald L. Castelino, Luke R. Bereznicki

**Affiliations:** 1 Unit for Medication Outcomes Research and Education, Division of Pharmacy, School of Medicine, Faculty of Health, University of Tasmania, Hobart, Tasmania, Australia; 2 Royal Hobart Hospital, Hobart, Tasmania, Australia; University of Brescia, ITALY

## Abstract

**Background:**

Adverse drug reactions (ADRs) are the major cause of medication-related hospital admissions in older patients living in the community. This study aimed to develop and validate a score to predict ADR-related hospitalization in people aged ≥65 years.

**Methods:**

ADR-related hospitalization and its risk factors were determined using a prospective, cross-sectional study in patients aged ≥65 years admitted to two hospitals. A predictive model was developed in the derivation cohort (n = 768) and the model was applied in the validation cohort (n = 240). ADR-related hospital admission was determined through expert consensus from comprehensive reviews of medical records and patient interviews. The causality and preventability of the ADR were assessed based on the Naranjo algorithm and modified Schumock and Thornton criteria, respectively.

**Results:**

In the derivation sample (mean [±SD] age, 80.1±7.7 years), 115 (15%) patients were admitted due to a definite or probable ADR; 92.2% of these admissions were deemed preventable. The number of antihypertensives was the strongest predictor of an ADR followed by presence of dementia, renal failure, drug changes in the preceding 3 months and use of anticholinergic medications; these variables were used to derive the ADR prediction score. The predictive ability of the score, assessed from calculation of the area under the receiver operator characteristic (ROC) curve, was 0.70 (95% confidence interval (CI) 0.65–0.75). In the validation sample (mean [±SD] age, 79.6±7.6 years), 30 (12.5%) patients’ admissions were related to definite or probable ADRs; 80% of these admissions were deemed preventable. The area under the ROC curve in this sample was 0.67 (95% CI 0.56–0.78).

**Conclusions:**

This study proposes a practical and simple tool to identify elderly patients who are at an increased risk of preventable ADR-related hospital admission. Further refinement and testing of this tool is necessary to implement the score in clinical practice.

## Introduction

Advancing age contributes to increased drug usage in older patients, which in turn is associated with an increased risk of adverse drug reactions (ADRs), causing significant morbidity and mortality [1]. The prevalence of ADRs in older outpatient clinic attendees ranges from 5–35% [2, 3]. ADRs are also one of the main reasons for hospitalization in older patients living in the community [4]. The proportion of all hospital admissions due to ADRs has ranged from 6–12% among older patients [1, 4–7]. While individual risk factors for ADRs have been identified [6, 8], health professionals are not able to easily identify elderly community-dwelling outpatients who are at high risk of being hospitalized due to an ADR. More than half of ADR-related hospitalizations are considered preventable [9].

In recent years, risk prediction models for ADRs in elderly patients have begun to emerge, offering practitioners a potential tool to assist clinical and therapeutic decision making, and facilitate targeting of additional resources toward this high-risk group [10, 11]. These tools were developed for use in secondary care hospital settings to help identify the risk of ADRs occurring during hospitalization. To our knowledge there is no prediction score available that has been developed for use in elderly patients with hospitalization due to ADR (as opposed to ADRs that arise during hospitalization) as the endpoint [12]. A tool developed that focussed on ADRs as a cause of hospitalization could potentially be used in primary care and at the point of hospital discharge to prioritize primary care-based medication management services to prevent ADR-related morbidity and mortality in patients at the highest risk of such events. We aimed to develop and validate a prediction model for ADR-related hospitalization in patients aged ≥65 years.

## Methods

### Derivation of a Score to Predict ADR-related Hospitalization

To develop the score [PADR-EC (Prediction of Hospitalization due to Adverse Drug Reactions in Elderly Community-Dwelling Patients) score], a prospective cross-sectional study was conducted at the Royal Hobart Hospital (RHH), which is the major public acute care hospital in Southern Tasmania. The study was approved by the Tasmanian Health and Medical Human Research Ethics Committee, and study participants provided their written informed consent to participate in the study. A convenience sample of all acute, unplanned, emergency admissions of patients aged ≥65 years admitted to medical wards over a period of 12 months (March 2014 to March 2015) were enrolled in the study. Patients were excluded if they were unwilling to participate, unable to be interviewed due to health or other reasons, or if their medical notes were not available for further investigation. The medical records of all consenting patients were reviewed within 48 hours of admission, and patients were interviewed as soon as practical after admission. Data collected included demographics, comorbidities, indicators of physical function and cognitive status, clinical diagnoses at admission, medications and medication changes prior to admission, previously documented ADRs, function in activities of daily living, social supports and living status. Patients and/or their relatives who were interviewed provided information about alcohol consumption, smoking status, recent hospital admissions, recent drug changes, drug allergies, use of over-the-counter (OTC) and herbal medicines, use of dosage administration aids, ADR occurrence within the last 3 months, regular pharmacy visits, and receipt of a Home Medicines Review (HMR), where a pharmacist conducts an interview with the patient regarding their medications and provides a report back to the general practitioner.

#### Predictive Variables for ADR-related Hospitalization

Medications taken prior to admission were coded according to the Anatomical Therapeutic and Chemical codes [13]. Calculation of the number of medications was based on the number of active ingredients [6], where the active ingredients in combination products were also available as single-ingredient products. Clinical diagnoses and comorbidities were coded according to the International Classification of Primary Care, 2^nd^ edition [14]. Comorbidity was measured using the Charlson comorbidity index (CCI) [15]. Renal failure was defined as an estimated glomerular filtration rate (eGFR) of less than 60 mL/min/1.73m^2^ [16]. Liver disease was defined as synthetic liver dysfunction or liver injury with raised transaminases greater than twice the normal range, or documented liver disease [17]. Anaemia was defined as a hemoglobin concentration below 120 g/L in women and below 130 g/L in men [18]. All comorbidities were defined as present if documented in the medical records. Functional independence was measured using the Barthel index [19]. Potentially inappropriate medications (PIMs) were identified using the updated Beers criteria [20]. Each class of PIMs within the Beers criteria was individually assessed. Recent drug changes prior to hospital admission were determined. Recent drug change was defined as addition of a new drug or deletion of an existing drug (excluding ‘when required’ medications) or a change in drug doses in the 3 months preceding the patient’s admission [21].

#### Identifying and Assessing the Presence of ADR-related Hospitalization

An ADR was defined as “a response which is noxious and unintended, and which occurs at doses normally used in humans for the prophylaxis, diagnosis, or therapy of disease, or for the modification of physiological function” [22]. We assessed every consenting patient during the study period to determine if the admission had been caused by an ADR. The reasons for hospitalization are multifactorial in many cases [23], and therefore the determination of whether a certain drug/drugs may have caused or contributed to an acute admission was based on comprehensive review of medical records and interview with the patient/relatives about their medication usage, including recent changes to drug therapy. The patients were interviewed in presence of their family members and the response to specific questions (S1 Table) were again verified using electronic patient file or digital medical records which contains all patient previous admission/discharge details. Comprehensive review of medical records included detailed review of medical and nursing records, medical record notes from primary care when available, medication reconciliation notes from clinical pharmacists, and an assessment of laboratory and other relevant clinical investigations. Patients were categorized as having an ADR if the cause of admission was consistent with the known adverse effect profile of the drug (according to Australian Medicines Handbook, or UpToDate database)[24, 25], if there was a temporal relation with the start of drug therapy and if, after appropriate investigations, other causes were excluded [26]. The clinical description of each ADR and the potential drug cause was collected, and the causality, preventability and eventual outcome of the suspected ADR-related hospitalization were assessed. We also classified ADRs as Type A and Type B reactions, based on Rawlins and Thompson [27]. Type A reactions were defined as dose-dependent and predictable from the known pharmacologic action of the drug and Type B reactions if otherwise.

The Naranjo algorithm was used to assess the causality of the relation between drug use and hospitalization [28]. ADRs were classified as definite (9–12 points), probable (5–8 points), possible (1–4 points), or doubtful (0 points). Only definite and probable ADRs that provoked hospitalization were considered for this study. ADRs observed during the hospital stay were excluded. The preventability of the ADR-related hospitalization was assessed using the modified Schumock and Thornton criteria [29, 30]. These criteria included (1) the drugs were not appropriate for the patient’s condition, (2) the dose, frequency and route of administration were inappropriate for the patient’s age, weight or disease state, (3) therapeutic drug monitoring or other necessary laboratory test was not performed, (4) the patient had a history of allergy or previous reaction to the administered drug, (5) a documented drug interaction was involved in the ADR, (6) a serum concentration above the therapeutic range was documented, (7) non-compliance was involved in the ADR or (8) a medication error was the cause of adverse reaction. The ADR-related hospitalization was considered to be preventable when it met any of these criteria.

**Fig 1:**
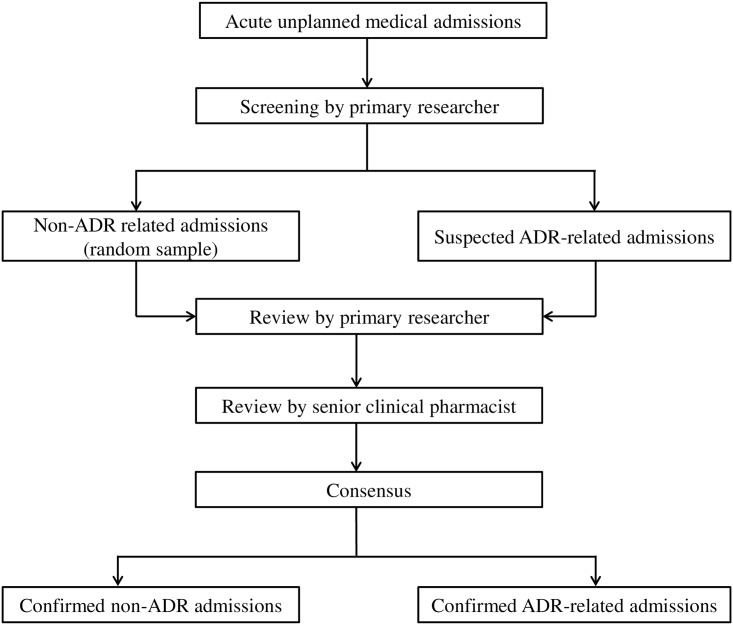
Diagram outlining the screening process of ADR-related hospital admission.

All patients initially categorized as having an ADR-related admission, and a random selection of 10% of cases without a suspected ADR-related admission, were independently and blindly assessed by a senior clinical pharmacist for the presence and classification of an ADR-related admission. The primary researcher and the senior clinical pharmacist met to reach a consensus decision on the presence of an ADR-related admission and excluded doubtful cases. The cases thought to involve an ADR-related admission were also assessed blindly by the clinical pharmacist reviewer for causality, severity and preventability. This review process had been used previously in similar studies [26, 31, 32]. The screening process and identification of ADR-related hospital admissions is outlined in Fig 1.

#### Statistical analysis

The PADR-EC score was developed in a similar manner to that described by Onder et al [11]. Clinically relevant variables that are easily applied and practical for use in a primary care setting were considered. The chi-square test or Fisher’s exact test were used to compare characteristics of those who experienced an ADR and those who did not. Variables identified as being associated with an ADR in the univariate analyses were entered into a binary logistic regression model. The variables with P values of <0.20 in the univariate analyses were candidates for inclusion in the binary logistic regression model, since more stringent significance levels can lead to the exclusion of potentially useful predictor variables [10, 33–35]. Multicollinearity between independent categorical variables was assessed using the phi coefficient [36]. When two variables had a phi coefficient ≥0.30, the model was trialled with each variable independently and the variable with higher predictive ability was entered into the final model. Variables retained in the final model were used to compute the PADR-EC score. A score of 1 was assigned to variables with an odds ratio (OR) between 1.00 and 1.49; a score of 2 to those with an OR between 1.5 and 2.49; a score of 3 to those with an OR between 2.5 and 3.49; a score of 4 to those with an OR between 3.5 and 4.49 and a score of 5 to those with an OR between 4.5 and 5.49. The PADR-EC score was computed based on the sum of scores of individual variables. Receiver operator characteristic (ROC) curves were constructed and area under the curve (AUC) calculated to determine the predictive ability of the PADR-EC score. Analyses were performed using SPSS version 20.0 (SPSS Inc, Chicago, Illinois).

### Validation study

In order to validate the PADR-EC score developed in the RHH sample (derivation stage), it was applied in a separate cohort of adults admitted to medical wards of the Launceston General Hospital (LGH). The LGH is the largest acute care facility and teaching hospital in the northern region of Tasmania. The study was approved by the Tasmanian Health and Medical Human Research Ethics Committee, and the study participants provided their written informed consent to participate in the study. Patients admitted to the LGH during the study period (September to December 2015) were enrolled according to the criteria discussed above. As before, data for all variables were recorded for each patient, along with details of any suspected ADR-related admission. To evaluate the predictive ability of the PADR-EC score, ROC curves were constructed and AUC calculated.

## Results

### Derivation of the PADR-EC Score

Over the 12-month study period, there were 5,027 acute unplanned medical admissions in patients aged ≥65 years at the RHH. Of the 1,271 (25%) patients screened during the study period, 503 (39.6%) were excluded either due to their unwillingness to consent (130 patients), or an inability to participate due to the severity of their medical condition, hearing impairment or low vision (373 patients). In total, 768 patients were included in the RHH cohort for final analysis. The characteristics of the study populations at the RHH and LGH are summarized in Table 1.

**Table 1:** Characteristics of the study populations in derivation and validation cohort.

Characteristics	Derivation stage (n = 768)	Validation stage (n = 240)
Age in years (mean ± SD)	80.1 ± 7.7	79.6 ± 7.6
Gender (n, %), Female	401 (52.2)	137 (57.1)
Number of medications before hospital admission (mean ± SD)	10.8 ± 5.2	9.9 ± 4.8
Number of comorbidities (mean ± SD)	5.5 ± 2.4	6.0 ± 2.5
**Living status (n, %)**		
Alone	308 (40.1)	101 (42.1)
With family or friends	433 (56.4)	135 (56.3)
Nursing home	27 (3.5)	4 (1.7)
**Comorbidities** (n, %)		
Dementia	54 (6.4)	15 (6.3)
Heart failure	136 (16.2)	52 (21.7)
Renal failure	406 (48.4)	125 (52.1)
Cerebrovascular disease	141 (16.8)	37 (15.4)
Diabetes	236 (28.1)	69 (28.8)
Chronic obstructive pulmonary disease	208 (24.8)	89 (37.1)
Cancer	179 (21.3)	67 (27.9)
Anemia	327 (39)	116 (48.3)
Liver disease	22 (2.6)	11 (4.6)
Depression	94 (11.2)	35 (14.6)
Hyperlipidemia	240 (28.6)	74 (30.8)
Ischemic heart disease	160 (19.1)	55 (22.9)
Vascular disease	309 (36.8)	90 (37.5)

Abbreviations: SD, standard deviation.

There was a consensus between the two expert reviewers in the majority of cases and, overall, 115 patients (15.0%) were judged as being admitted due to ADRs. There were 17 doubtful cases that were not classified as ADR-related admissions and were added to the control group (n = 653). There were 9 (5.8%) definite and 106 (69.3%) probable ADRs based on the Naranjo algorithm. Most of the ADR-related hospitalizations were considered preventable (106, 92.2%) and all ADRs were classified as Type A reactions except one which was considered as a Type B reaction. As seen in Table 2, univariate analysis identified that PIMs (anticholinergics, antiarrhythmics, benzodiazepines), PIMs use in dementia or cognitive impairment, hospital admission in the preceding month, hospital admission in the preceding 3 months, drug changes in the preceding 3 months, 9 or more regular medications, 7 or more comorbidities, renal failure, dementia, heart failure, anemia, vascular disease, Charlson comorbidity score ≥6, use of alcohol and age ≥85 years were associated with an increased risk of ADR-related hospital admission (P ≤0.20). Specific drug classes contributing to the risk of ADR-related admission included antihypertensives (1–2, ≥3), angiotensin converting enzyme inhibitors (ACEIs) or angiotensin receptor blockers (ARBs), beta-blockers, drugs of narrow therapeutic index (digoxin, amiodarone, theophylline, phenytoin, carbamazepine and sodium valproate), psycholeptics, benzodiazepines, tricyclic antidepressants, diuretics and tricyclic antidepressants or psycholeptics. The antihypertensives included alpha_2_-adrenergic agonists, alpha_1_-adrenoreceptor antagonists, beta-blockers, calcium channel blockers, diuretics, agents acting on the renin-angiotensin system and vasodilators. Fixed-dose antihypertensive combination therapy, including triple therapy, was used in 12% (n = 95) of patients. The PIMs (anticholinergics) included antihistamines (chlorpheniramine and promethazine), antispasmodics (hyoscyamine products and atropine products) and tertiary tricyclic antidepressants (amitriptyline, imipramine and doxepin >6 mg/day). When variables were excluded due to multicollinearity, the variables retained in the model were hospital admission in the preceding month, drug changes in the preceding 3 months, 7 or more comorbidities, renal failure, dementia, drugs of narrow therapeutic index, antihypertensives (1–2, ≥3), anemia, PIMs (anticholinergics), psycholeptics, age ≥85 years and use of alcohol. Binary logistic regression retained drug changes in the preceding 3 months, renal failure, dementia, antihypertensives (1–2, ≥3), and PIMs (anticholinergics) as significant predictors of ADR-related hospital admission (Table 3). These variables were assigned scores based on their respective ORs (Table 4).

**Table 2:** Characteristics of patients experiencing adverse drug reaction-related and non-adverse drug reaction-related hospital admissions at the Royal Hobart Hospital (n = 768).

Variable	Number (%)	Number (%)	P Value
	No ADR (n = 653)	ADR (n = 115)	
**Age (years)**			
65–84	454 (69.5)	70 (60.9)	0.07*
≥85	199 (30.5)	45 (39.1)	
**Gender**			
Male	316 (48.4)	51 (44.3)	0.42
Female	337 (51.6)	64 (55.7)	
**Drug-related variables**			
Use of OTC medications	275 (42.1)	45 (39.1)	0.55
Use of herbal medications	156 (23.9)	24 (20.9)	0.48
Drug changes in the preceding 3 months	304 (46.6)	70 (60.9)	0.01*
***Number of medications***			
0–8	245 (37.5)	32 (27.8)	0.05*
≥9	408 (62.5)	83 (72.2)	
***Inappropriate medications (Therapeutic category/drug)***			
Anticholinergics	57 (8.7)	21 (18.3)	0.002*
Benzodiazepines	128 (19.6)	29 (25.2)	0.17*
Antiarrhythmics	18 (2.8)	8 (7)	0.04*
Digoxin	10 (1.5)	2 (1.7)	0.70
Metoclopramide	14 (2.1)	4 (3.5)	0.33
***Inappropriate medications (Disease)***			
Heart failure	10 (1.5)	1 (0.9)	1.00
Dementia or cognitive impairment	14 (2.1)	8 (7)	0.01*
**Disease-related variables**			
***Charlson Comorbidity Index***			
0–5	344 (52.7)	52 (45.2)	0.14*
≥ 6	309 (47.3)	63 (54.8)	
≥7 Comorbidities	206 (31.5)	51 (44.3)	0.01*
Cerebrovascular diseases	117 (17.9)	24 (20.9)	0.45
Diabetes	200 (30.6)	36 (31.3)	0.89
Anemia	270 (41.3)	57 (49.6)	0.10*
Depression	81 (12.4)	13 (11.3)	0.74
Acute cognitive impairment	41 (6.3)	5 (4.3)	0.42
Dementia	42 (6.4)	12 (10.4)	0.12*
Renal failure ǂ	323 (49.7)	83 (72.8)	<0.001*
Liver disease	18 (2.8)	4 (3.5)	0.56
Heart failure	108 (16.5)	28 (24.3)	0.04*
COPD	176 (27)	32 (27.8)	0.85
Cancer	156 (23.9)	23 (20)	0.36
Hyperlipidemia	204 (31.2)	36 (31.3)	0.99
Ischemic heart disease	133 (20.4)	27 (23.5)	0.45
Vascular disease	254 (38.9)	55 (47.8)	0.07*
**Variables related to drug classes**			
Drugs of narrow therapeutic index	158 (24.2)	38 (33)	0.05*
Antithrombotics	444 (68)	85 (73.9)	0.21
***Antihypertensives***			
0	131 (20.1)	6 (5.2)	
1–2	328 (50.2)	51 (44.3)	<0.001*
≥3	194 (29.7)	58 (50.4)	
ACEIs or ARBs	357 (54.7)	77 (67)	0.01*
Calcium channel blockers	172 (26.3)	34 (29.6)	0.47
Cardiac glycosides	69 (10.6)	15 (13)	0.43
Beta-blockers	223 (34.2)	52 (45.2)	0.02*
Drugs used in diabetes	156 (23.9)	29 (25.2)	0.76
NSAIDs	27 (4.1)	6 (5.2)	0.60
Opioids	199 (30.5)	41 (35.7)	0.27
Psycholeptics	158 (24.2)	36 (31.3)	0.11*
Antipsychotics	37 (5.7)	9 (7.8)	0.37
Benzodiazepines	137 (21)	32 (27.8)	0.10*
Tricyclic antidepressants	57 (8.7)	15 (13)	0.14*
Tricyclic antidepressants or psycholeptics	197 (30.2)	46 (40)	0.04*
Antiplatelets	320 (49)	61 (53)	0.42
Diuretics	309 (47.3)	80 (69.6)	<0.001*
Antibacterials	94 (14.4)	14 (12.2)	0.53
Anticoagulants	140 (21.4)	30 (26.1)	0.27
**Other variables**			
Admission in preceding month	159 (24.3)	38 (33)	0.05*
Admission in preceding 3 months	269 (41.2)	55 (47.8)	0.18*
Use of dosage administration aid	259 (39.7)	45 (39.1)	0.91
Use of generics †	345 (60.5)	62 (62.6)	0.69
Use of alcohol	241 (36.9)	35 (30.4)	0.18*
Smokers	72 (11)	9 (7.8)	0.30
Presence of ADR within 3 months ♀	106 (16.5)	22 (20.2)	0.35
Previous ADR ǂ	389 (59.8)	65 (57)	0.57
Regular pharmacy visits	582 (89.1)	105 (91.3)	0.48
HMR in the preceding 3 months	41 (6.3)	5 (4.3)	0.42
Assistance required with ≥1 activity of daily living	433 (66.3)	83 (72.2)	0.22
Albumin <3.5 g/dL	293 (44.9)	54 (47)	0.68
Falls	38 (5.8)	6 (5.2)	0.80

Abbreviations: OTC, over-the-counter; COPD, chronic obstructive pulmonary disease; ACEIs, angiotensin converting enzyme inhibitors; ARBs, angiotensin receptor blockers; NSAIDs, non-steroidal anti-inflammatory drugs; ADR, adverse drug reaction; HMR, Home Medicines Review.

*P value ≤0.20.

^ǂ^ 4 participants had missing values.

^†^ 99 participants had missing values.

^♀^ 17 participants had missing values.

**Table 3:** Binary logistic regression of factors associated with adverse drug reaction-related hospital admission in the derivation study at the Royal Hobart Hospital (n = 768).

Variable	Adjusted OR (95% CI)	P value
Age ≥85 years	1.33 (0.86–2.06)	0.20
Drug changes in the preceding 3 months	1.54 (1.00–2.37)	0.05
Anemia	1.08 (0.70–1.65)	0.74
Renal failure	1.97 (1.22–3.17)	0.01
Drugs of narrow therapeutic index	1.15 (0.73–1.81)	0.55
Dementia	2.44 (1.17–5.10)	0.02
Admission in preceding month	1.31 (0.82–2.07)	0.26
Number of comorbidities ≥7	1.07 (0.69–1.66)	0.76
Number of antihypertensives		
1–2	3.00 (1.22–7.38)	0.02
≥3	4.75 (1.89–11.93)	0.001
Anticholinergics	2.09 (1.16–3.75)	0.01
Psycholeptics	1.24 (0.78–1.98)	0.36
Use of alcohol	0.80 (0.51–1.25)	0.32

Abbreviations: CI, confidence interval; OR, odds ratio.

**Table 4:** Variables included in the risk score.

Variable	OR (95% CI)	Points
Drug changes in the preceding 3 months	1.54 (1.00–2.37)	2
Renal failure	1.97 (1.22–3.17)	2
Dementia	2.44 (1.17–5.10)	2
Number of antihypertensives		
1–2	3.00 (1.22–7.38)	3
≥3	4.75 (1.89–11.93)	5
Anticholinergics	2.09 (1.16–3.75)	2

Abbreviations: CI, confidence interval; OR, odds ratio.

Drug changes in the preceding 3 months, renal failure, dementia and PIMs (anticholinergics) were scored at 2 points and antihypertensives received a score of 3 points (1–2 antihypertensive agents) or 5 points (≥3 antihypertensives). The range of scores was from 0 to 11, with a median of 5 (IQR 5). The area under the ROC curve, which assesses the ability of the risk score to predict ADR-related hospitalization in the whole population, was 0.70 (95% CI 0.65–0.75) (S1 Fig). A score cut off at 6 provided a good balance between sensitivity (72.2%) and specificity (58.0%). The risk of patients having an ADR-related hospitalization was more than three times higher in those who scored ≥6 compared to those who scored <6 (OR 3.59 [95% CI 2.32–5.55]).

### Validation study

Over the study period of 4 months, 518 patients were screened at the LGH. Of these, 123 patients were excluded due to their unwillingness to consent and 155 patients could not be recruited due to the severity of their medical condition. In total, 240 patients were included in the LGH cohort for the validation of the PADR-EC score. Definite (2, 5.2%) and probable (28, 73.7%) ADR-related hospital admissions were observed in 30 patients (12.5%) in this sample. The patients’ characteristics are summarized in Table 1.

**Fig 2:**
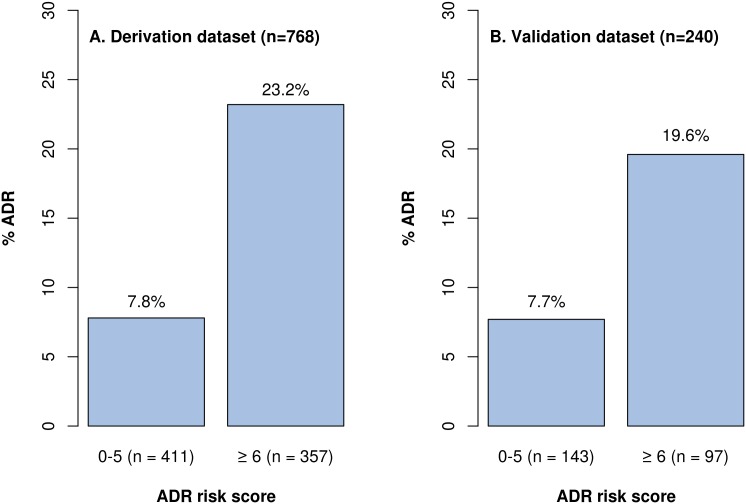
Adverse drug reaction rate according to risk score. (A) The adverse drug reaction rate at cut off score at 6 in the derivation dataset at the Royal Hobart Hospital. (B) The adverse drug reaction rate at cut off score at 6 in the validation dataset at the Launceston General Hospital.

The majority of the ADR-related hospitalizations were considered preventable (24, 80%). When the PADR-EC score was applied to the LGH data set, the AUC was 0.67 (95% CI 0.56–0.78) (S1 Fig). A score cut off at 6 provided a good balance between sensitivity (63%) and specificity (63%). In the LGH data set, patients who scored ≥6 had almost three times the risk of ADR-related hospitalization compared to those scoring <6 (OR 2.92 [95% CI 1.32–6.46]). The percentage increase of ADR-related hospitalization with respect to the cut-off score ≥6 in both the RHH and LGH data set is outlined in Fig 2.

## Discussion

We developed and validated a simple and robust approach to identifying community-dwelling elderly patients at risk of hospitalization due to ADRs. To our knowledge, this is the first study to develop such a score. This score has the potential to assist healthcare practitioners to identify those elderly patients for whom intervention may reduce the risk of ADRs and subsequent hospitalization. In addition, patients could be stratified at hospital discharge according to their risk of subsequent admission of an ADR, and appropriate medication management services provided accordingly post-discharge.

The variables we identified as independent predictors of ADRs in the elderly have been described in previous studies [37–40]. Drug changes in the preceding 3 months were found to predict ADR-related admission in the present study. Changes in medications such as using a different dose, discontinuing therapies, and taking new drugs were observed in the majority of elderly patients after hospital discharge in a follow-up study [21] and clinically significant changes to medicines or treatment plans within the last 3 months were found to be a risk factor for medication-related problems [37, 41]. ADRs attributed to medication changes occurred in 20% of patients during transfer from hospital and nursing home in a study conducted in United States [42]. Renal failure was found to be another significant predictor of ADR-related admission in the present study. The relation between renal insufficiency and ADRs is well documented in the literature [43, 44]. Impaired renal function (OR 2.6; 95% CI 1.6–4.2) was found to be a major determinant of preventable medication-related hospital admission in a prospective multicentre study [45]. Renal failure was also found to be predictor of ADRs in the GerontoNet ADR score study (OR 1.2; 95% CI 0.9–1.5) as well as in a prospective study of elderly individuals who presented to an emergency department (OR 1.5; 95% CI 1.1–2.2) [11, 46]. In the present study, we found elderly patients with renal failure were twice as likely to be hospitalized due to ADRs. The presence of dementia was identified as another independent predictor of ADR-related hospitalization. The prevalence of ADRs in elderly patients with dementia was 5.0% in another study; half of the ADRs were due to use of psychotropic and anti-dementia drugs [47]. Drug-related problems appeared to be responsible for the majority of hospitalizations among old people with dementia and the most common drug-related problem was an ADR (18.7%) in a recent study [32].

PIMs are significantly associated with ADRs and subsequent hospital admission [12]. Inappropriate medications were associated with a two-fold increased risk of an ADR in the elderly in a prospective study [17]. Among the PIMs, use of anticholinergics was found to be the predictor of ADR-related hospital admission in the present study. The prevalence of exposure to anticholinergic medicines in the elderly has ranged from 22.8% to 55.9% [48]. The use of drugs with anticholinergic adverse effects is often inappropriate in older patients aged ≥65 years [49]. Many age-related and disease-related conditions may predispose older patients to ADRs related to anticholinergic drugs [50]. These drugs have many effects in the elderly, ranging from dry mouth and constipation to confusion, delirium and severe cognitive impairment [51]. Another important predictor of ADR-related admission in the present study was the use of multiple antihypertensives. Approximately 70% of patients with hypertension require two or more drugs to achieve their target blood pressure [52]. The use of multiple antihypertensives in elderly patients is a frequent cause of hospital admission [53]. Antihypertensives were found to be one of the most frequently implicated drugs in causing ADRs and the risk is higher with combination therapy and in patients receiving multiple antihypertensive drugs [54–56]. In a community-based randomized open label trial [57] there was a significant increase in dizziness reported with combination antihypertensive therapy. The prevalence of orthostatic hypotension has been reported to be between 35% and 65% for the elderly and is mainly associated with the use of antihypertensive medications [58–60].

This study found that almost 15% of admissions in the elderly were due to ADRs. Meta-analysis of observational studies in the elderly reported a similarly high rate (16.6%) of ADR-related hospitalization [61]. The PADR-EC score has a predictive ability of 70% to discriminate patients who are at high risk of ADR-related hospitalization and those who are not. The subsequent validation study found a predictive ability of 67%. The sensitivity of the score was 72% in the derivation cohort, which indicates the ability of the score to correctly classify a patient as a victim of preventable ADR-related admission. Importantly, the expert reviewers also identified that the majority of ADR-related admissions were preventable. The predictive ability of the risk score could therefore be utilized to identify ADRs which may be prevented by monitoring of patients’ drug therapy, addressing inappropriate dosing, aiding patient compliance with therapy and managing drug interactions to avoid subsequent admission. To our knowledge, there is no previously developed ADR prediction tool for use in primary care to which to compare the present study findings; however, the score performed comparably to validated ADR prediction tools used to predict ADR risk in hospitalized patients, such as those developed by Onder et al. (predictive ability of 71%) [11] and Tangiisuran et al. (74%) [10]. While there are tools available to predict the risk of emergency admission to hospital [62–64], our tool was developed to focus specifically on the risk hospitalisation due to an ADR to enable primary health care professionals to better direct medication management services to prevent ADRs.

The PADR-EC score consists of five clinical variables that are easy to apply and practical to assess in elderly patients. The development of this tool may assist general practitioners or primary care physicians in identifying older patients who have a high risk of ADRs and subsequent emergency hospital admissions [12]. This is particularly important considering that it may be challenging for healthcare practitioners to easily identify patients who are at risk of hospitalization due to ADRs, partly due to significant time pressures in office-based practice [65]. The PADR-EC score could potentially be integrated into prescribing software to alert primary care physicians to their patients’ risk of ADRs and prompt appropriate preventive measures. Such preventive measures may include medication review, avoiding use of PIMs, computer-based prescribing systems and comprehensive geriatric assessment [66], as well as deprescribing (withdrawal of an inappropriate medication) [67] and avoiding unnecessary polypharmacy when drugs are no longer efficacious or beneficial, or when safer alternatives exist [68]. The PADR-EC score could also be applied at hospital discharge to identify older patients who are at higher risk of admission for ADRs to facilitate post-discharge medication management review services and/or closer monitoring by relevant health professionals to prevent subsequent hospitalization. Thus, application of PADR-EC score could potentially play a role in reducing the risk of ADR-related hospitalizations in the elderly.

Limitations include the score’s specificity of 58%, resulting in a chance of incorrectly labelling patients as ‘having an ADR risk’ who may not be at risk (false positives). The validation sample had almost equal or slightly less discriminatory power compared to the derivation sample, suggesting that further refinement and testing of the PADR-EC score is required before implementing the score in clinical practice. The time restraints did not allow us to validate the tool further in a community setting to follow the patients in primary care to observe an ADR related hospital admission outcome versus other disease related outcomes. However, the variables used to derive the score only used patient information before hospital admission and all the patients recruited in the study were primary care patients. There were inherent limitations in assessing the predictor ‘drug changes in the preceding 3 months’, as this could have potentially been influenced by incomplete records. We minimized this issue by interviewing the patient/relatives about their medication usage, including recent changes to drug therapy, and comprehensively reviewing medical records and, importantly, medication reconciliation reports from clinical pharmacists. Some of the variables arose from patient interviews and thus could be a subject to recall bias; this was limited by conducting interviews in the presence of family members. We could not recruit some patients due to difficulty in getting the consent mainly due to their severity of disease. Thus, obtaining consent was the limiting step, and a retrospective study could have included these additional patients, although such a study would lose the ability to obtain information through interview. The degree of generalizability of the proposed model is restrained by convenience sampling, however the large sample size used in the study may have reduced the sampling error and hence, may not have affected the applicability of the proposed model. We cannot assess the time frame for ADR occurrence after identifying patients at risk. Hence, further studies are required to address this issue in order to predict ADRs and subsequent admission in a timely fashion. Further investigation is needed to determine the absolute risk for people identified as being at high-risk of ADRs using this score, and whether interventions in these patients by health care professionals are able to reduce this risk.

## Conclusion

We propose a simple, efficient and practical tool to identify elderly patients living in the community who are at increased risk of ADR-related hospitalization. The PADR-EC score was developed and externally validated reasonably in a cohort of elderly subjects admitted to two participating centres. It has the potential to be easily used, mainly by primary care physicians to identify elderly patients vulnerable to ADRs, and target interventions to prevent subsequent hospitalization. Even though further refinement and testing of this tool is necessary before implementing the score in clinical practice, this tool could provide a useful starting point to predict risk for ADR-related hospitalization in the elderly. Further studies are required to assess the clinical utility of this tool in different settings and populations.

## Supporting Information

S1 Fig Receiver operator characteristic curve for the derivation dataset (A) and the validation dataset (B). The area under the curve are 0.70 (95% CI 0.65–0.75) and 0.67 (95% CI 0.56–0.78) for the derivation and validation datasets respectively.
(TIF)

S1 Table Questionnaires to participants. (DOCX)

## References

[1] Franceschi M, Scarcelli C, Niro V, Seripa D, Pazienza AM, Pepe G, et al Prevalence, clinical features and avoidability of adverse drug reactions as cause of admission to a geriatric unit: a prospective study of 1756 patients. Drug Saf. 2008;31(6):545–56. 18484788 10.2165/00002018-200831060-00009

[2] Gurwitz JH, Field TS, Harrold LR, Rothschild J, Debellis K, Seger AC, et al Incidence and preventability of adverse drug events among older persons in the ambulatory setting. JAMA. 2003;289(9):1107–16. 12622580 10.1001/jama.289.9.1107

[3] Hanlon JT, Schmader KE, Koronkowski MJ, Weinberger M, Landsman PB, Samsa GP, et al Adverse drug events in high risk older outpatients. J Am Geriatr Soc. 1997;45(8):945–8. 9256846 10.1111/j.1532-5415.1997.tb02964.x

[4] Marcum ZA, Amuan ME, Hanlon JT, Aspinall SL, Handler SM, Ruby CM, et al Prevalence of unplanned hospitalizations caused by adverse drug reactions in older veterans. J Am Geriatr Soc. 2012;60(1):34–41. 10.1111/j.1532-5415.2011.03772.x 22150441 PMC3258324

[5] Mannesse CK, Derkx FH, de Ridder MA, Man in 't Veld AJ, van der Cammen TJ. Contribution of adverse drug reactions to hospital admission of older patients. Age Ageing. 2000;29(1):35–9. 10690693 10.1093/ageing/29.1.35

[6] Wawruch M, Zikavska M, Wsolova L, Kuzelova M, Kahayova K, Strateny K, et al Adverse drug reactions related to hospital admission in Slovak elderly patients. Arch Gerontol Geriatr. 2009;48(2):186–90. 10.1016/j.archger.2008.01.004 18313773

[7] Conforti A, Costantini D, Zanetti F, Moretti U, Grezzana M, Leone R. Adverse drug reactions in older patients: an Italian observational prospective hospital study. Drug Healthc Patient Saf. 2012;4:75–80. 10.2147/DHPS.S29287 22888275 PMC3413040

[8] Onder G, Pedone C, Landi F, Cesari M, Della Vedova C, Bernabei R, et al Adverse drug reactions as cause of hospital admissions: results from the Italian Group of Pharmacoepidemiology in the Elderly (GIFA). J Am Geriatr Soc. 2002;50(12):1962–8. 12473007 10.1046/j.1532-5415.2002.50607.x

[9] Chan M, Nicklason F, Vial JH. Adverse drug events as a cause of hospital admission in the elderly. Intern Med J. 2001;31(4):199–205. 11456032 10.1046/j.1445-5994.2001.00044.x

[10] Tangiisuran B, Scutt G, Stevenson J, Wright J, Onder G, Petrovic M, et al Development and validation of a risk model for predicting adverse drug reactions in older people during hospital stay: Brighton Adverse Drug Reactions Risk (BADRI) model. PLoS One. 2014;9(10):e111254 10.1371/journal.pone.0111254 25356898 PMC4214735

[11] Onder G, Petrovic M, Tangiisuran B, Meinardi MC, Markito-Notenboom WP, Somers A, et al Development and validation of a score to assess risk of adverse drug reactions among in-hospital patients 65 years or older: the GerontoNet ADR risk score. Arch Intern Med. 2010;170(13):1142–8. 10.1001/archinternmed.2010.153 20625022

[12] Parameswaran Nair N, Chalmers L, Peterson GM, Bereznicki BJ, Castelino RL, Bereznicki LR. Hospitalization in older patients due to adverse drug reactions -the need for a prediction tool. Clin Interv Aging. 2016;11:497–505. 10.2147/CIA.S99097 27194906 PMC4859526

[13] Pahor M, Chrischilles EA, Guralnik JM, Brown SL, Wallace RB, Carbonin P. Drug data coding and analysis in epidemiologic studies. Eur J Epidemiol. 1994;10(4):405–11. 7843344 10.1007/BF01719664

[14] Classification Committee of the World Organization of Family Doctors (WICC) 1998 ICPC-2: International Classification of Primary Care. 2nd edn Oxford: Oxford University Press.

[15] Charlson M, Szatrowski TP, Peterson J, Gold J. Validation of a combined comorbidity index. J Clin Epidemiol. 1994;47(11):1245–51. 7722560 10.1016/0895-4356(94)90129-5

[16] Levey AS, Bosch JP, Lewis JB, Greene T, Rogers N, Roth D. A more accurate method to estimate glomerular filtration rate from serum creatinine: a new prediction equation. Modification of Diet in Renal Disease Study Group. Ann Intern Med. 1999;130(6):461–70. 10075613 10.7326/0003-4819-130-6-199903160-00002

[17] O'Connor MN, Gallagher P, Byrne S, O'Mahony D. Adverse drug reactions in older patients during hospitalisation: are they predictable? Age Ageing. 2012;41(6):771–6. 10.1093/ageing/afs046 22456465

[18] World Health Organization. Nutritional Anaemias: Report of a WHO Scientific Group. Geneva, Switzerland: World Health Organization; 1968. Technical report series No. 405. 4975372

[19] Mahoney FI, Barthel DW. Functional Evaluation: The Barthel Index. Md State Med J. 1965;14:61–5. 14258950

[20] American Geriatrics Society updated Beers Criteria for potentially inappropriate medication use in older adults. J Am Geriatr Soc. 2012;60(4):616–31. 10.1111/j.1532-5415.2012.03923.x 22376048 PMC3571677

[21] Cochrane RA, Mandal AR, Ledger-Scott M, Walker R. Changes in drug treatment after discharge from hospital in geriatric patients. BMJ. 1992;305(6855):694–6. 1393119 10.1136/bmj.305.6855.694 PMC1882955

[22] World Health Organization. International Drug Monitoring: The Role of the Hospital. Report of a WHO Meeting. Geneva, Switzerland: World Health Organization; 1969. Technical Report Series No. 425. Available: http://apps.who.int/iris/handle/10665/40747. Accessed 2014 March. 4981079

[23] Gustafsson M, Sjolander M, Pfister B, Jonsson J, Schneede J, Lovheim H. Drug-related hospital admissions among old people with dementia. Eur J Clin Pharmacol. 2016;72(9):1143–53. 10.1007/s00228-016-2084-3 27377393

[24] Australian Medicines Handbook 2015 (online). Adelaide: Australian Medicines Handbook Pty Ltd; 2015 January. Available: http://amhonline.amh.net.au/. Accessed 2015 March.

[25] UpToDate. A Comprehensive Clinical Database. Wolters Kluwer, Alphen aan den Rijn, the Netherlands. Available: http://www.uptodate.com. Accessed 2014–2015.

[26] Pirmohamed M, James S, Meakin S, Green C, Scott AK, Walley TJ, et al Adverse drug reactions as cause of admission to hospital: prospective analysis of 18 820 patients. BMJ. 2004;329(7456):15–9. 10.1136/bmj.329.7456.15 15231615 PMC443443

[27] Rawlins MD, Thompson JW. Mechanisms of adverse drug reactions In: Davies DM, ed. Textbook of adverse drug reactions. Oxford: Oxford University Press, 1991:18–45.

[28] Naranjo CA, Busto U, Sellers EM, Sandor P, Ruiz I, Roberts EA, et al A method for estimating the probability of adverse drug reactions. Clin Pharmacol Ther. 1981;30(2):239–45. 7249508 10.1038/clpt.1981.154

[29] Calderon-Ospina C, Bustamante-Rojas C. The DoTS classification is a useful way to classify adverse drug reactions: a preliminary study in hospitalized patients. Int J Pharm Pract. 2010;18(4):230–5. 10.1111/j.2042-7174.2010.00039.x 20636675

[30] Schumock GT, Thornton JP. Focusing on the preventability of adverse drug reactions. Hosp Pharm. 1992;27(6):538 10118597

[31] Gurwitz JH, Field TS, Avorn J, McCormick D, Jain S, Eckler M, et al Incidence and preventability of adverse drug events in nursing homes. Am J Med. 2000;109(2):87–94. 10967148 10.1016/s0002-9343(00)00451-4

[32] Gustafsson M, Sjolander M, Pfister B, Jonsson J, Schneede J, Lovheim H. Drug-related hospital admissions among old people with dementia. Eur J Clin Pharmacol. 2016. 10.1007/s00228-016-2084-3 27377393

[33] Min SY, Park DW, Yun SC, Kim YH, Lee JY, Kang SJ, et al Major predictors of long-term clinical outcomes after coronary revascularization in patients with unprotected left main coronary disease: analysis from the MAIN-COMPARE study. Circ Cardiovasc Interv. 2010;3(2):127–33. 10.1161/CIRCINTERVENTIONS.109.890053 20407112

[34] May S, Gardiner E, Young S, Klaber-Moffett J. Predictor Variables for a Positive Long-Term Functional Outcome in Patients with Acute and Chronic Neck and Back Pain Treated with a McKenzie Approach: A Secondary Analysis. J Man Manip Ther. 2008;16(3):155–60. 10.1179/jmt.2008.16.3.155 19119405 PMC2582422

[35] In Lee K, Koval JJ. Determination of the best significance level in forward stepwise logistic regression. Communications in Statistics—Simulation and Computation. 1997;26(2):559–75.

[36] Muir SW, Berg K, Chesworth B, Klar N, Speechley M. Balance impairment as a risk factor for falls in community-dwelling older adults who are high functioning: a prospective study. Phys Ther. 2010;90(3):338–47. 10.2522/ptj.20090163 20056721

[37] Kaufmann CP, Stampfli D, Hersberger KE, Lampert ML. Determination of risk factors for drug-related problems: a multidisciplinary triangulation process. BMJ Open. 2015;5(3):e006376 10.1136/bmjopen-2014-006376 25795686 PMC4368979

[38] SHPA Committee of Specialty Practice in Clinical Pharmacy. SHPA. Standards of Practice for Clinical Pharmacy Services. J Pharm Pract Res 2005; 35: 122–46.

[39] Wu C, Bell CM, Wodchis WP. Incidence and economic burden of adverse drug reactions among elderly patients in Ontario emergency departments: a retrospective study. Drug Saf. 2012;35(9):769–81. 10.2165/11599540-000000000-00000 22823502 PMC3714138

[40] Hajjar ER, Hanlon JT, Artz MB, Lindblad CI, Pieper CF, Sloane RJ, et al Adverse drug reaction risk factors in older outpatients. Am J Geriatr Pharmacother. 2003;1(2):82–9. 15555470 10.1016/s1543-5946(03)90004-3

[41] SHPA Committee of Specialty Practice in Clinical Pharmacy. SHPA. Standards of Practice for Clinical Pharmacy Services. J Pharm Pract Res 2005; 35: 122–46.

[42] Boockvar K, Fishman E, Kyriacou CK, Monias A, Gavi S, Cortes T. Adverse events due to discontinuations in drug use and dose changes in patients transferred between acute and long-term care facilities. Arch Intern Med. 2004;164(5):545–50. 10.1001/archinte.164.5.545 15006832

[43] Hellden A, Bergman U, von Euler M, Hentschke M, Odar-Cederlof I, Ohlen G. Adverse drug reactions and impaired renal function in elderly patients admitted to the emergency department: a retrospective study. Drugs Aging. 2009;26(7):595–606. 10.2165/11315790-000000000-00000 19655826

[44] Corsonello A, Pedone C, Corica F, Mussi C, Carbonin P, Antonelli Incalzi R. Concealed renal insufficiency and adverse drug reactions in elderly hospitalized patients. Arch Intern Med. 2005;165(7):790–5. 10.1001/archinte.165.7.790 15824299

[45] Leendertse AJ, Egberts AC, Stoker LJ, van den Bemt PM. Frequency of and risk factors for preventable medication-related hospital admissions in the Netherlands. Arch Intern Med. 2008;168(17):1890–6. 10.1001/archinternmed.2008.3 18809816

[46] Chen YC, Fan JS, Chen MH, Hsu TF, Huang HH, Cheng KW, et al Risk factors associated with adverse drug events among older adults in emergency department. Eur J Intern Med. 2014;25(1):49–55. 10.1016/j.ejim.2013.10.006 24200546

[47] Laroche ML, Perault-Pochat MC, Ingrand I, Merle L, Kreft-Jais C, Castot-Villepelet A, et al Adverse drug reactions in patients with Alzheimer's disease and related dementia in France: a national multicentre cross-sectional study. Pharmacoepidemiol Drug Saf. 2013;22(9):952–60. 10.1002/pds.3471 23794320

[48] Salahudeen MS, Hilmer SN, Nishtala PS. Comparison of anticholinergic risk scales and associations with adverse health outcomes in older people. J Am Geriatr Soc. 2015;63(1):85–90. 10.1111/jgs.13206 25597560

[49] Ness J, Hoth A, Barnett MJ, Shorr RI, Kaboli PJ. Anticholinergic medications in community-dwelling older veterans: prevalence of anticholinergic symptoms, symptom burden, and adverse drug events. Am J Geriatr Pharmacother. 2006;4(1):42–51. 10.1016/j.amjopharm.2006.03.008 16730620

[50] Feinberg M. The problems of anticholinergic adverse effects in older patients. Drugs Aging. 1993;3(4):335–48. 8369593 10.2165/00002512-199303040-00004

[51] Tune LE. Anticholinergic effects of medication in elderly patients. J Clin Psychiatry. 2001;62 Suppl 21:11–4. 11584981

[52] Frank J. Managing hypertension using combination therapy. Am Fam Physician. 2008;77(9):1279–86. 18540493

[53] Butt TF, Branch RL, Beesley L, Martin U. Managing hypertension in the very elderly: effect of adverse drug reactions (ADRs) on achieving targets. J Hum Hypertens. 2010;24(8):514–8. 10.1038/jhh.2009.116 20130597

[54] Jatau AI, Aung MM, Kamauzaman TH, Rahman AF. Prevalence of Drug-Related Emergency Department Visits at a Teaching Hospital in Malaysia. Drugs Real World Outcomes. 2015;2(4):387–95. 10.1007/s40801-015-0045-2 26689834 PMC4674517

[55] Olsen H, Klemetsrud T, Stokke HP, Tretli S, Westheim A. Adverse drug reactions in current antihypertensive therapy: a general practice survey of 2586 patients in Norway. Blood Press. 1999;8(2):94–101. 10451036 10.1080/080370599438266

[56] Rende P, Paletta L, Gallelli G, Raffaele G, Natale V, Brissa N, et al Retrospective evaluation of adverse drug reactions induced by antihypertensive treatment. J Pharmacol Pharmacother. 2013;4(Suppl 1):S47–50. 10.4103/0976-500X.120954 24347982 PMC3853669

[57] Everett BM, Glynn RJ, Danielson E, Ridker PM. Combination therapy versus monotherapy as initial treatment for stage 2 hypertension: a prespecified subgroup analysis of a community-based, randomized, open-label trial. Clin Ther. 2008;30(4):661–72. 18498915 10.1016/j.clinthera.2008.04.013

[58] Mets TF. Drug-induced orthostatic hypotension in older patients. Drugs Aging. 1995;6(3):219–28. 7620234 10.2165/00002512-199506030-00005

[59] Poon IO, Braun U. High prevalence of orthostatic hypotension and its correlation with potentially causative medications among elderly veterans. J Clin Pharm Ther. 2005;30(2):173–8. 10.1111/j.1365-2710.2005.00629.x 15811171

[60] Mussi C, Ungar A, Salvioli G, Menozzi C, Bartoletti A, Giada F, et al Orthostatic hypotension as cause of syncope in patients older than 65 years admitted to emergency departments for transient loss of consciousness. J Gerontol A Biol Sci Med Sci. 2009;64(7):801–6. 10.1093/gerona/glp028 19349588

[61] Beijer HJ, de Blaey CJ. Hospitalisations caused by adverse drug reactions (ADR): a meta-analysis of observational studies. Pharm World Sci. 2002;24(2):46–54. 12061133 10.1023/a:1015570104121

[62] Hippisley-Cox J, Coupland C. Predicting risk of emergency admission to hospital using primary care data: derivation and validation of QAdmissions score. BMJ Open. 2013;3(8):e003482 10.1136/bmjopen-2013-003482 23959760 PMC3753502

[63] Shelton P, Sager MA, Schraeder C. The community assessment risk screen (CARS): identifying elderly persons at risk for hospitalization or emergency department visit. Am J Manag Care. 2000;6(8):925–33. 11186504

[64] Lyon D, Lancaster GA, Taylor S, Dowrick C, Chellaswamy H. Predicting the likelihood of emergency admission to hospital of older people: development and validation of the Emergency Admission Risk Likelihood Index (EARLI). Fam Pract. 2007;24(2):158–67. 10.1093/fampra/cml069 17210987

[65] Cutler DM, Everett W. Thinking outside the pillbox—medication adherence as a priority for health care reform. N Engl J Med. 2010;362(17):1553–5. 10.1056/NEJMp1002305 20375400

[66] Onder G, van der Cammen TJ, Petrovic M, Somers A, Rajkumar C. Strategies to reduce the risk of iatrogenic illness in complex older adults. Age Ageing. 2013;42(3):284–91. 10.1093/ageing/aft038 23537588

[67] Reeve E, Gnjidic D, Long J, Hilmer S. A systematic review of the emerging definition of 'deprescribing' with network analysis: implications for future research and clinical practice. Br J Clin Pharmacol. 2015;80(6):1254–68. 10.1111/bcp.12732 27006985 PMC4693477

[68] Lavan AH, Gallagher P. Predicting risk of adverse drug reactions in older adults. Ther Adv Drug Saf. 2016;7(1):11–22. 10.1177/2042098615615472 26834959 PMC4716390

