# Supplementary figures and images for: Prediction of Hospitalization due to Adverse Drug Reactions in Elderly Community-Dwelling Patients (The PADR-EC Score)
Source: PLoS One. 2016 Oct 31;11(10):e0165757. doi: 10.1371/journal.pone.0165757 (PMC5087856; doi:10.1371/journal.pone.0165757)

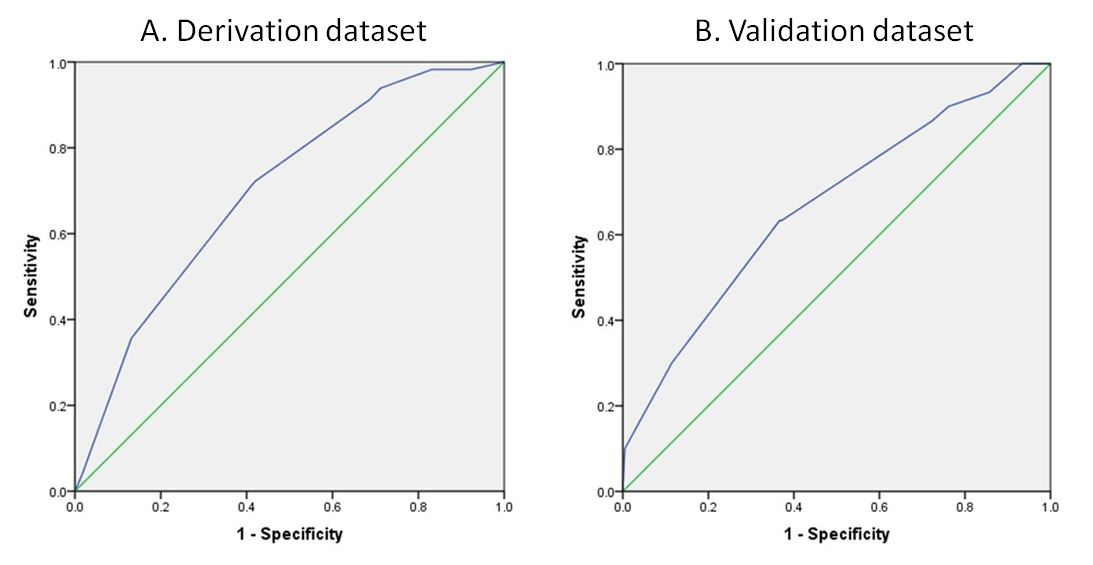

Supplement: S1 Fig — The area under the curve are 0.70 (95% CI 0.65–0.75) and 0.67 (95% CI 0.56–0.78) for the derivation and validation datasets respectively. (TIF) [file pone.0165757.s001.tif]
