# Supplementary material for: Prediction of Hospitalization due to Adverse Drug Reactions in Elderly Community-Dwelling Patients (The PADR-EC Score)
Source: PLoS One. 2016 Oct 31;11(10):e0165757. doi: 10.1371/journal.pone.0165757 (PMC5087856; doi:10.1371/journal.pone.0165757)
Supplement: S1 Table — (DOCX) [file pone.0165757.s002.docx]

| **No.** | **Questionnaire** | **Answer** |
| --- | --- | --- |
| 1 | a) Do you drink alcohol?  If answer “YES”,  b) What type of alcohol do you like to drink (e.g. beer, wine, spirits)?  c) In a week, how many times would you drink and how much would you drink each time? | Yes No |
| 2 | Do you smoke? | Yes No |
| 3 | Were you recently hospitalised for any reason? If so, when? | Yes No |
| 4 | Do you remember any recent changes in drug therapy before your admission at Royal Hobart Hospital?  If answer “YES”, please recollect the names of the medicines changed? | Yes No |
| 5 | Are you allergic to any medicines – e.g. Penicillin or Aspirin?  If “YES”, list the allergy and the reaction | Yes No |
| 6 | Within the last 3 months, have you experienced any ‘bad reactions’ to any medicines? | Yes No |
| 7 | Have you taken any OTC medications apart from your regular medications?  If “YES”, please recollect the names/s of those | Yes No |
| 8 | Do you take any complimentary medicines/herbal medicines?    If “YES”, please recollect the names/s of those? | Yes No |
| 9 | Do you have a regular community pharmacy?  How many pharmacies do you attend for your prescription medications? | Yes No |
| 10 | Do you usually use dosage administration aids (for example, a dosette box, or Websterpak, where the pharmacy packs your medicines into days and weeks for you)? | Yes No |
| 11 | a) Do you use different brands of the same medication, sometimes called generic prescription medications? | Yes No |
| 12 | Have you recently had a Home Medicines Review (pharmacist interviewing you about your medications in your home)/MedsCheck/Diabetes MedsCheck? | Yes No |

**S1 Table. Questionnaires to participants**
